# An automated method for determining the cytoadhesion of *Plasmodium falciparum*-infected erythrocytes to immobilized cells

**DOI:** 10.1186/s12936-015-0632-4

**Published:** 2015-03-14

**Authors:** Casper Hempel, Ida M Boisen, Akinwale Efunshile, Jørgen AL Kurtzhals, Trine Staalsø

**Affiliations:** Centre for Medical Parasitology at Department of Clinical Microbiology, Copenhagen University Hospital Department Clinical Microbiology, 7602, Ole Maaløesvej 26, 2200 Copenhagen N, Denmark; Department of International Health, Immunology and Microbiology, University of Copenhagen, Copenhagen, Denmark; Institute of Medical Microbiology and Infectious Disease Epidemiology, Medical Faculty, University of Leipzig, Leipzig, Germany; Department of Medical Microbiology, Federal Teaching Hospital/Ebonyi State University, Abakaliki, Nigeria

**Keywords:** *Plasmodium falciparum*, Cytoadhesion, Chinese hamster ovary cells, Endothelial cells, Field isolates

## Abstract

**Background:**

*Plasmodium falciparum* exports antigens to the surface of infected erythrocytes causing cytoadhesion to the host vasculature. This is central in malaria pathogenesis but *in vitro* studies of cytoadhesion rely mainly on manual counting methods. The current study aimed at developing an automated high-throughput method for this purpose utilizing the pseudoperoxidase activity of intra-erythrocytic haemoglobin.

**Methods:**

Chinese hamster ovary (CHO) cells were grown to confluence in chamber slides and microtiter plates. Cytoadhesion of co-cultured *P. falciparum*, selected for binding to CHO cells, was quantified by microscopy of Giemsa-stained chamber slides. In the automated assay, binding was quantified spectrophotometrically in microtiter plates after cell lysis using tetramethylbenzidine as peroxidase-catalysed substrate. The relevance of the method for binding studies was assessed using: i) binding of *P. falciparum*-infected erythrocytes to CHO cells over-expressing chondroitin sulfate A and ii) CHO cells transfected with CD36. Binding of infected erythrocytes including field isolates to primary endothelial cells was also performed. Data was analysed using linear regression and Bland-Altman plots.

**Results:**

The manual and automated quantification showed strong, positive correlation (r^2^ = 0.959, p <0.001) and with similar detection limit and precision. The automated assay showed the expected dose-dependent reduction in binding to CHO cells when blocking with soluble chondroitin sulfate A or anti-CD36 antibody. Quantification of binding to endothelial cells showed clear distinction between selected vs. non-selected parasite lines. Importantly, the assay was sufficiently sensitive to detect adhesion of field isolates to endothelial cells.

**Conclusions:**

The assay is simple and in a reproducible manner quantifies erythrocyte adhesion to several types of immobilized cells.

## Background

Malaria is a major cause of morbidity and mortality, particularly in developing countries. The species *Plasmodium falciparum* accounts for the majority of fatal malaria infections. A central part of pathogenesis and a major determinant for a complicated infection is the parasites’ ability to adhere to endothelial cells in order to avoid splenic clearance [1-3]. Cytoadhesion results in activation of endothelial cells, impaired microcirculation, hypoxia, inflammation and pro-coagulant phenotype [4-6].

Adhesion assays and quantification of the binding of infected erythrocytes to endothelial cells are in demand to study parasite binding patterns [3,7] and to study the consequences binding has for endothelial cells [4,8,9]. The majority of binding assays are static binding assays and quantification of infected erythrocytes bound to adherent cells is performed by using the gold standard, manual counting of Giemsa-stained erythrocytes [7,10]. Microscopical examination is quite labour-intensive and also prone to subjectivity. Other ways to quantify cytoadhesion include the use of radioactive ^3^H-hypoxanthine incorporation assays [8]. Recently, a computer-assisted approach to quantification of infected erythrocytes was developed but when applied to parasites bound to endothelial cells this method was associated with considerable variation [11].

The aim of the study was to develop an automated, high-throughput assay that was reliable and simple. For this purpose, the presence of high amounts of haem in erythrocytes was utilized due to its pseudoperoxidase characteristics [12,13]; an aspect that can give rise to high background staining in immunohistochemistry [12]. Parasite binding was quantified by using Chinese hamster ovary (CHO) cells expressing ligands that malaria parasites have affinity to [3,7,10]. The method was compared with examination by microscopy and showed to have similar precision and limit of detection. The method was subsequently tested using primary, human endothelial cells and clinical parasite isolates.

## Methods

### *Plasmodium falciparum* culture, panning and synchronization

*Plasmodium falciparum* FCR3 and 3D7 were cultured *in vitro* according to standard protocols [14]. Briefly, the parasites were grown in culture flasks at 37°C at 4% haematocrit in HEPES-buffered RPMI 1640 medium (Gibco, Life Technologies, Paisley, UK) supplemented with 5 mg/ml Albumax II (Life Technologies), 0.02 mg/ml hypoxanthine (Sigma-Aldrich, MO, USA), 0.05 mg/ml gentamycin (Gibco, Life Technologies), and 0.18 mg/ml L-glutamine (Sigma-Aldrich) in an atmosphere of 2% O_2_, 5.5% CO_2_ and 92.5% N_2_. Subculture with the addition of blood group O erythrocytes was done throughout the study. Human blood was obtained with verbal informed consent from healthy volunteers, a procedure that is permitted without ethical approval from the Ethics Committee in the Capital Region of Denmark. Field isolates were cultured as described above although culture medium was additionally supplemented with 2% human serum. Field isolates were from patients with uncomplicated malaria.

Prior to binding studies, parasite cultures were synchronized to late stage (late trophozoites and schizonts) by gelatine flotation [15]. Gelatine flotation was carried out for 20 min at 37°C in sterile-filtered 0.75% gelatine (Gibco, Life Technologies) dissolved in HEPES-buffered RPMI-1640 medium (Gibco, Life Technologies). Parasite stage and parasitaemia were determined by microscopy of Giemsa-stained thin blood smears.

### CHO cell culture

Four different types of CHO cells were used: CHO-K1 (wild type, CCL-61, American Type Culture Collection (ATCC), Manassas, VA, USA), CHO-CD36 (transfected with human CD36, CRL-2092, ATCC), CHO-A745 (pgsA-745, xylosyltransferase deficient; does not produce glycosaminoglycans (GAG), CRL-2242, ATCC) and CHO-D677 (pgsD-677, negative for heparan sulfate expression, CRL-2244, ATCC). CHO-K1 and CHO-A745 were used as negative controls for CD36- and chondroitin sulfate (CS)-specific binding, respectively. CHO cells were cultured in HEPES-buffered RPMI-1640 medium (Gibco, Life Technologies) supplemented with 10% foetal bovine serum (Gibco, Life Technologies) and 0.05 mg/ml gentamycin (Gibco, Life Technologies).

### Culture of human brain microvascular endothelial cells (hBMEC) and human aortic endothelial cells (hAEC)

Primary hBMEC (ScienCell Research laboratories, CA, US) and primary hAEC (ATCC) were cultured in endothelial cell medium (ScienCell Research Laboratories) supplemented with 5% FBS, endothelial cell growth supplement (ScienCell Research Laboratories) and Penicillin (100 U/ml) and streptomycin (100 μg/ml) (ScienCell Research Laboratories). hBMEC were cultured on a Fibronectin matrix (Sigma-Aldrich). For hBMEC passage 5-7 was used, while for hAEC passage 4-7 was used in the binding assays.

### Selecting *Plasmodium falciparum* for binding to immobilized cells

Parasites were selected for binding to either CHO-D677 or to CHO-CD36 as previously described [16]. Parasites selected for binding to endothelial cells was performed similarly. Briefly, gelatine-purified parasites were first negatively selected by co-incubating with gamma-irradiated CHO cells in Albumax-enriched culture medium for 60 min. FCR3 parasites selected for CD36 binding were negatively selected on CHO-K1 while parasites selected for CS-A binding were incubated on CHO-A745 cells. The infected erythrocytes not binding CHO cells were then positively selected by transferring them to the CHO cell line of interest (CHO-CD36 and CHO-D677, respectively). After 30 min the culture flasks were washed and unbound cells removed. Infected erythrocytes binding to the CHO cells (positive selection) were left to co-incubate over night at 37°C. The next day ring-stage parasites were collected and cultured as described above. Panning was repeated at least five times and binding efficiency was notably increased during the process. FCR3 parasites selected for binding to human CS proteoglycan isolated from placental tissue were also used [17]. 3D7 parasites positively selected to human bone marrow endothelial cells (ATCC). After five rounds of selection cytoadhesion was notably increased compared with unselected 3D7 parasites. 3D7 parasites selected for expression of variant surface antigens associated with severe malaria [18] bound poorly to primary endothelial cells and were used as control. Clinical isolates were not selected prior to binding studies.

### Binding and quantification of binding to immobilized cells

Binding experiments were performed in either eight-well Labtek chamber slides (NUNC, Thermo Fischer, Denmark) for microscopy or optical 96-well plates with polymer base (NUNC) for automated counting. CHO cells were seeded at a concentration of 1.5 × 10^5^ cells/ml in the wells two days prior to binding experiments in order to obtain a confluent monolayer. On the day of the experiment, *P. falciparum* cultures were gelatine-purified and the late stage parasites were added to the wells and left to co-incubate on an orbital shaker for 45 min at 37°C. Then, 1.25 × 10^6^ erythrocytes in 100 μl medium were added to each well of the 96-well plates and 2.8 × 10^6^ erythrocytes in 224 μl to the chamber slides corresponding to an area of 31 sq mm in 96-well plates and 70 sq mm in the chamber slides. This resulted in approximately 50:1 ratio between erythrocytes and CHO cells in both plate types. The parasitaemia after gelatine purification was in the range 40-50% in the experiments. After incubation, plates were inverted and unbound cells were removed by a 30-min gravity-wash in 2% FBS-supplemented PBS at 37°C, as previously described [11]. To assess lower detection limit erythrocyte concentrations were decreased in some studies.

Cells in the eight-well chamber slides were fixed with methanol and stained with 1% Giemsa for 30 min in 1/15 M Sørensen’s phosphate buffer (pH = 7.4). The number of erythrocytes binding to CHO cells was quantified by microscopy at 1,000x magnification with a reticule in the eyepiece corresponding to field of view (FOV) equal to 0.01 sq mm. The number of fields needed for a precise count of bound erythrocytes was determined experimentally by counting an increasing number of areas covered by the reticule. The number of FOV used was 16. The random selection of FOV was compared with a systematic uniform random sampling (SURS) method using a 2-D fractionator [19]. SURS was performed as a 4 × 4 matrix and compared with a similar number of areas but randomly selected. When performing the SURS, a random starting point was selected and from that particular point there was 100 μm between the areas counted both in the y and x direction. All microscopy was carried out on blinded specimens.

Cells in 96-well plates were permeabilized with 100 μl 0.1% Triton X-100 (Sigma-Aldrich) in PBS for 15 min at room temperature. Then, 100 μl tetramethylbenzidine (TMB, R&D Systems, Oxon, UK) was added to each well, with some left blank as controls, and the plate was incubated for up to 5 min covered in aluminium foil. The reaction was stopped by the addition of 50 μl 1M sulphuric acid per well. The optical density was measured at 450 nm using 540 nm as correcting wavelength on a microplate reader (Multiscan EX, Thermo Labsystems, Thermo Fischer, MA, USA). For the dynamic assays, absorbance was measured 1 min after applying TMB and thereafter with set time intervals at 650 nm. All quantifications were made in quadruplicate and all experiments were repeated at least twice.

A standard curve was generated for the automated quantification in order to calculate the number of bound erythrocytes. The standard was made from the same gelatine-enriched parasite culture as used in the experiment. The cells were counted and lysed with Triton x-100 (Sigma-Aldrich) as described above. For comparison, uninfected erythrocytes, unsynchronized infected erythrocytes and the lower phase of gelatine flotation (negative selection for late stage parasites) were tested to assess if parasite stage affected pseudoperoxidase activity.

To address whether the method was also applicable for using human endothelial cells and parasites selected for binding as well as field isolates the same procedures were followed as described for CHO cells above.

### Inhibition of binding using anti-CD36 and CS-A

CS-A (Sigma-Aldrich) and anti-CD36 antibody (clone FA6.152, Beckman Coulter, Brea, CA, USA) were applied to address if the binding was specific and if it could be inhibited. CS-A was incubated with gelatine-purified infected erythrocytes at 37°C on an orbital shaker for 30 min at the concentrations described prior to performing the actual binding experiments. Anti-CD36 was incubated with CD36-expressing CHO cells on an orbital shaker at 37°C for 30 min at the concentrations described prior to the actual binding experiments. Uninfected erythrocytes were included as controls when comparing the binding of CHO cell-selected parasites to different types of CHO cells.

### Quantification of binding to hBMEC and hAEC

Binding experiments were performed and quantified as described above. In these studies both a laboratory strain (3D7), one CS proteoglycan-selected FCR3 strain and Nigerian field isolates were included. Field isolates were obtained with consent from children with uncomplicated malaria in Ilero, Nigeria [20]. One laboratory strain had been selected for binding to human bone marrow endothelial cells (five rounds) and compared with 3D7 parasites [18].

### Statistical analyses

Normally distributed data with similar variance were analysed by one-way ANOVA followed by *post hoc* t tests with Holm correction. Bland-Altman plots were performed according to the literature [21]. Correlation analyses between the two methods for quantifying cytoadhesion were performed using Pearson’s correlation. Statistical analyses were carried out using R version 2.15.2 [22] and p <0.05 was considered statistical significant.

## Results

### Erythrocytes have peroxidase activity and this activity is maintained upon *Plasmodium falciparum* infection

**Figure 1:**
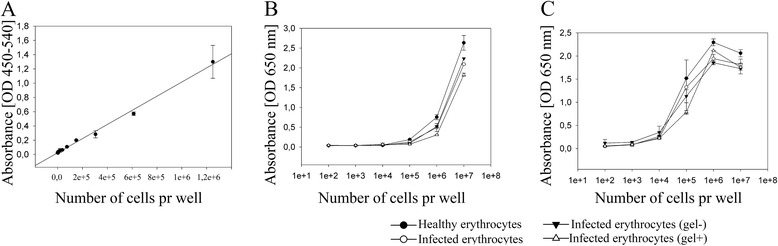
**Intraerythrocytic haem acts as a pseudoperoxidase and can be quantified with a chromogenic substrate. A)** Lysed *P. falciparum*-infected erythrocytes oxidize the chromogenic substrate TMB. There is a strong correlation between the OD-value and number of lysed iRBC (r^2^ = 0.98, p <0.001). **B)** The chromogenic signals generated from lysates of known numbers of erythrocytes and infected erythrocytes from different conditions were compared 1 min after addition of TMB. No statistical difference was observed between erythrocyte populations (p >0.7). See text for details. **C)** When the haem-driven reaction was continued 11 min the signal decreased in wells with the highest numbers of erythrocytes and increased in wells with low number of erythrocytes. On all graphs, symbols represent mean values and error bars show standard deviation.

Haem in erythrocytes can act as a pseudoperoxidase [12] and chromogenic substrates have previously been used to quantify haem [13]. The chromogenic signal correlated strongly with the number of lysed erythrocytes (Figure 1A, r^2^ = 0.98, p <0.001). When comparing the signal generated from lysed uninfected erythrocytes with lysates from infected erythrocytes (mixed stage), ring-stage erythrocytes (gel-) or late-stage erythrocytes (gel+) no difference could be detected (Figure 1B, p >0.7).

Leaving the oxidation of TMB to run for longer time (t = 11 min) did not result in differences between uninfected erythrocytes and the different preparations of infected erythrocytes (Figure 1C, p >0.2). However, the signal intensity dropped in lysates with the highest number of erythrocytes.

### No improvement of manual counting results when applying SURS

The intention was to quantify TMB oxidation for determining the number of erythrocytes adherent to the CHO-cells in the wells. First, an attempt was made to improve the manual counting method in order to optimize this as gold standard for the comparison with the automated method. Random selection of FOV was compared with SURS in the microscopic assay. No difference in mean value (p = 0.3) or coefficient of variation (p = 0.4) was observed and it was decided to use random selection of FOV in the rest of the study.

### The automated assay can detect the same quantitative changes in binding as microscopic examination

**Figure 2:**
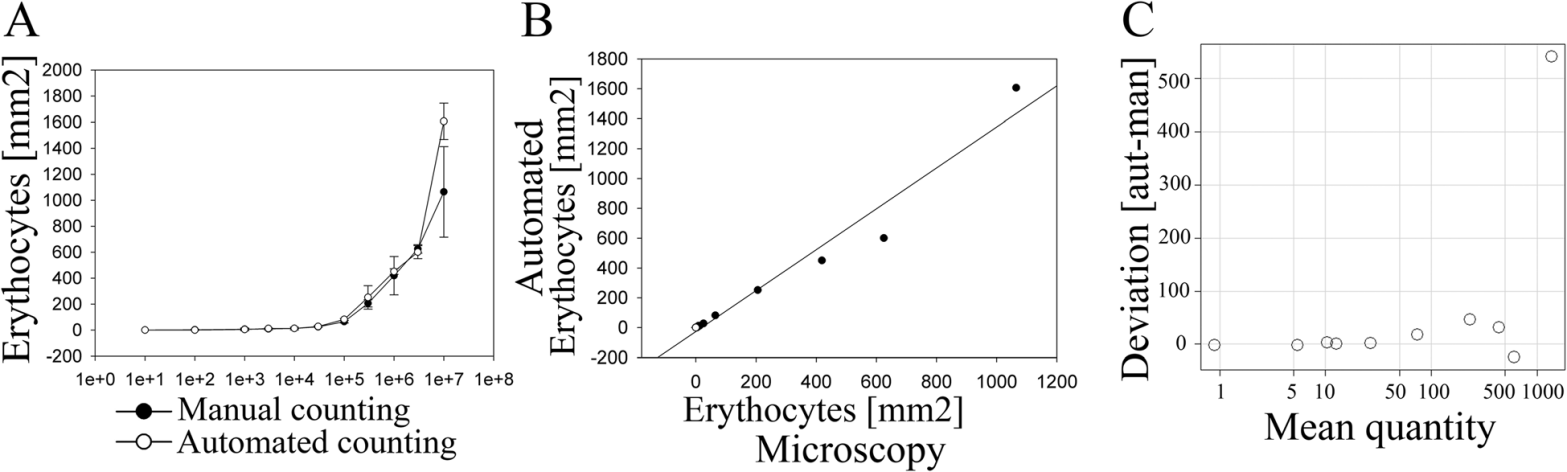
**Automated counting can detect binding comparable to manual microscopic examination. A)** Increasing densities of parasites were added to wells and the numbers of adherent cells were quantified using both microscopic examination and automated detection. **B)** The mean value for each tested density for both methods was plotted against each other. The quantifications were highly correlated (r = 0.98, p <0.001). For the regression line, erythrocyte counts were calculated from OD-values in the automated method using a standard curve. **C)** Bland-Altman plot from a single experiment. Each circle represents the difference in mean quantity of adherent cells when counts from manual, microscopic counting are subtracted from automated reading at different seeding densities. 0 on the y-axis denotes identical result using both methods.

In order to be routinely used and comparable with manual counting the automated method should have the same precision, accuracy and lower detection limit as the manual method. Different concentrations of infected erythrocytes were added to CHO cells and the quantity of bound cells was determined. The detection limit of the automated assay was 10^5^ gelatine-enriched erythrocytes. At this load the automated assay detected a significantly higher OD-value as compared with CHO cells only (Figure 2A, p = 0.004). Similarly, the lower detection limit with the manual counting assay was 10^5^ applied erythrocytes (Figure 2A, p = 0.003). These detection limits correspond to approximately 80-100 erythrocytes per sq mm in both the chamber slides and the 96-well plates. There seems to be deviant detection at high parasite densities but it remains statistically insignificant (p = 0.1).

The methods gave highly correlated results (Figure 2B, r^2^ = 0.959, p <0.001). In a Bland-Altman plot [21] difference between the two methods was plotted against the mean number of adherent erythrocytes per sq mm for the two methods (Figure 2C). Overall, the plot suggested very high degree of similarity between the two measures. However, at the highest density, the automated method tended to overestimate binding without being significantly different (p = 0.1).

### The automated assay can be used to quantify adhesive properties and adhesion blocking effects

**Figure 3:**
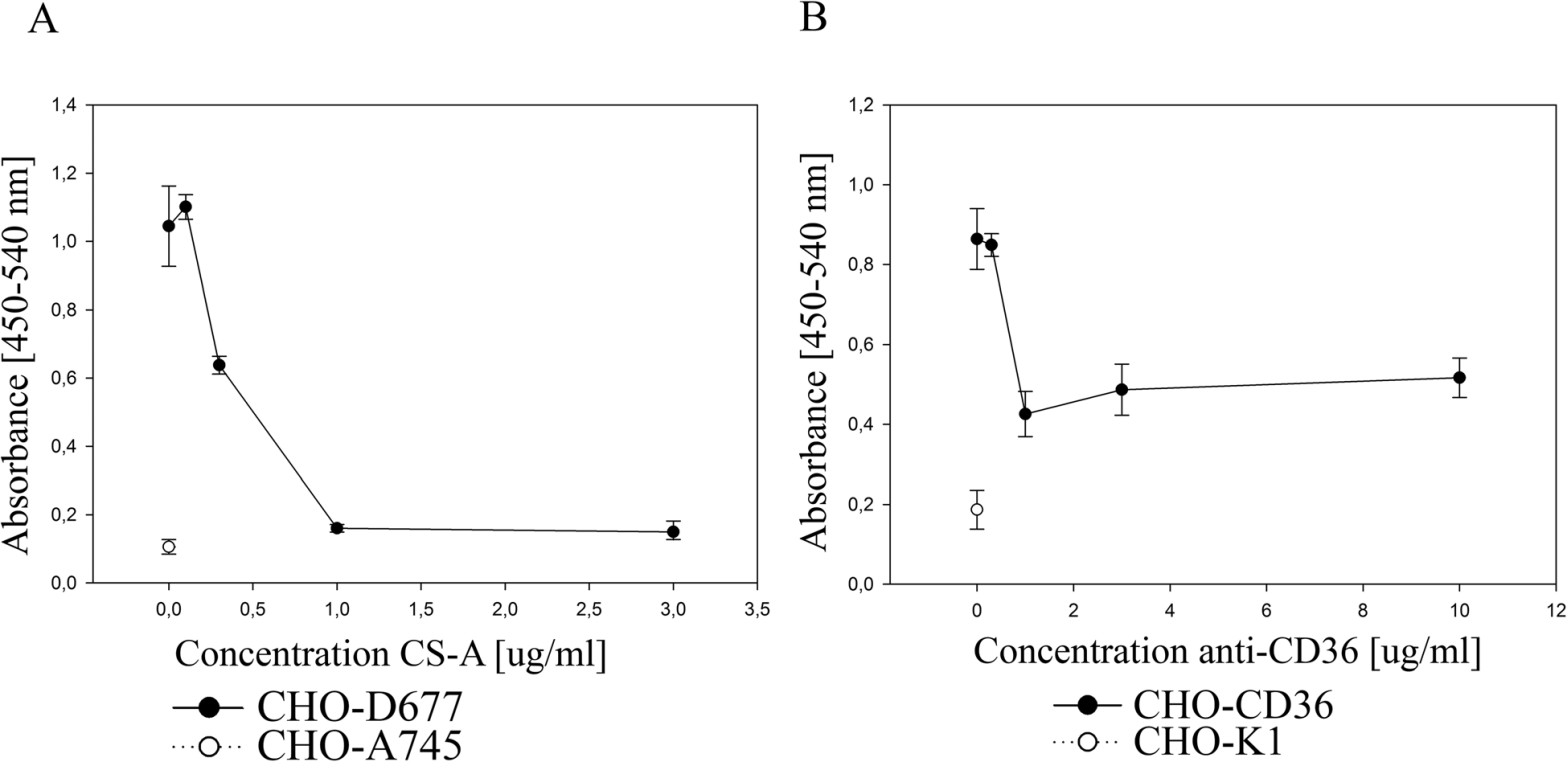
**Inhibition of binding can be assayed with high precision. A)** Dose-dependent inhibition of binding to CHO-D677 (only expressing CS) after addition of increasing concentrations of soluble CS-A. CHO-A745 was included as non-CS-A control. **B)** Dose-dependent inhibition of binding to CHO-CD36 (transfected with human CD36) after addition of increasing concentrations of anti-CD36 is added. CHO-K1 (wild type) was included as CD36-negative control. Error bars show standard deviation.

A precision comparable to manual counting prompted to test whether the automated assay was useful in adhesion blocking assays. Parasites selected for CS-A affinity were added to confluent CHO-D677 cells in the presence or absence of soluble CS-A. CS-A dose-dependently decreased binding as determined by the automated method (Figure 3A). Also, parasites selected for CD36 affinity were added to confluent CHO-CD36 cells and incubation with anti-CD36 dose-dependently decreased binding (Figure 3B).

### The automated method can be used to quantify binding of laboratory strains and field isolates to human endothelial cells

**Figure 4:**
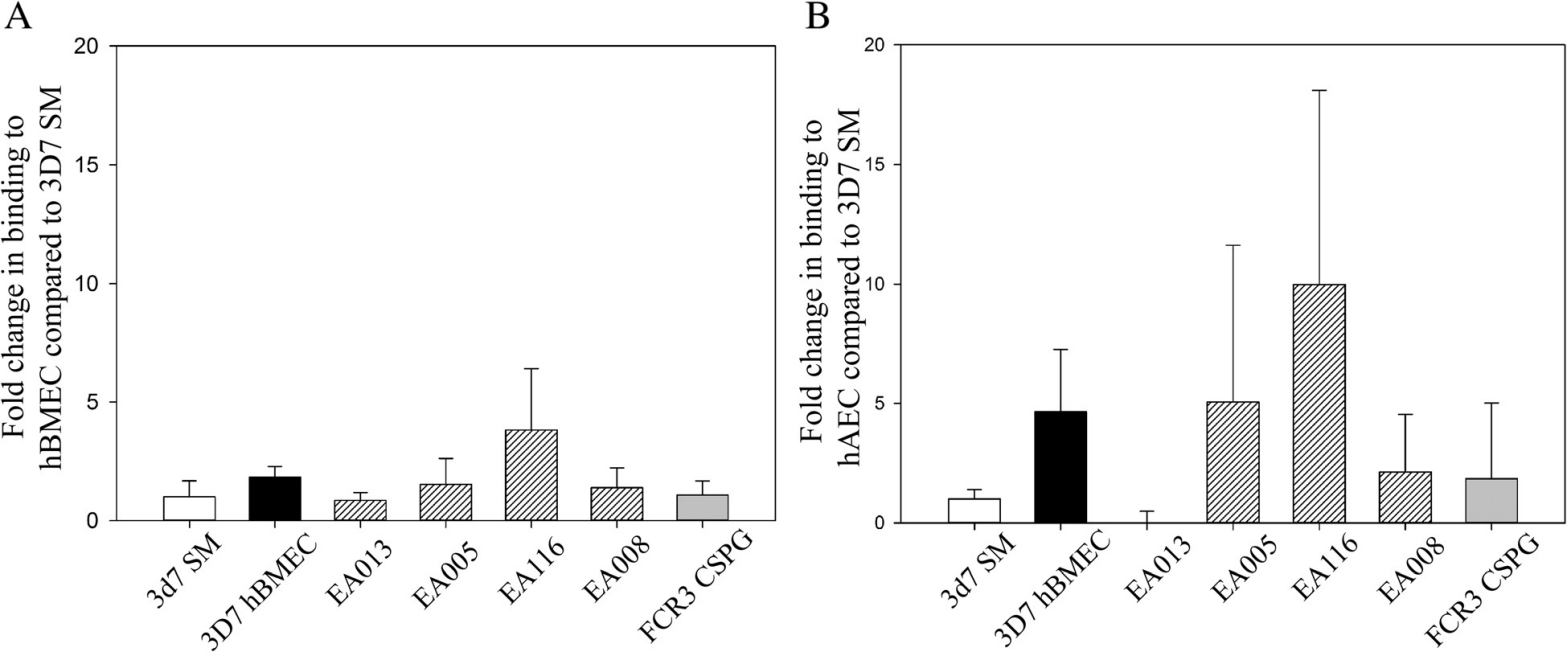
**Binding of field isolates to human endothelial cells as well as parasites pre-selected for endothelial cytoadhesion can be quantified.** Seven different parasite lines were tested for binding to hBMEC and hAEC. **A)** Bar graph showing binding to hBMEC. Binding to hBMEC is normalised to the binding of 3D7 SM [18] parasites not pre-selected to endothelial binding. **B)** Bar graph showing binding to hAEC. Binding to hAEC is normalised to the binding of 3D7 SM [18] parasites not pre-selected to endothelial binding. Bar show the mean fold change and error bars show standard deviation.

To further demonstrate the use of this method in cytoadhesion assays, hBMEC and hAEC were seeded as described for CHO cells. A laboratory strain (*P. falciparum* 3D7) selected for binding to endothelial cells was also used and binding compared with unselected 3D7 parasites as well as with a laboratory strain (FCR3) selected on human CS proteoglycan. A Nigerian field isolates was included to demonstrate that the method has sufficient sensitivity to detect cytoadhesion of unselected field isolates (Figure 4). The results demonstrate that selection on human bone marrow endothelial cells both improves binding to hBMEC (Figure 4A) and hAEC (Figure 4B). Interestingly, the method demonstrated that field isolates have highly variable cytoadhesive properties (Figure 4).

## Discussion

The aim was to develop a simple, objective and unbiased method for quantifying the binding of *P. falciparum* to immobilized cells. By using different CHO cell lines and malaria-infected erythrocytes selected for either CS or CD36 binding it was investigated if endogenous peroxidase activity of the infected erythrocytes was a suitable marker to quantify adherent erythrocytes. The present results demonstrate that this method had the same detection level as manual counting using microscopy of Giemsa-stained chamber slides.

Erythrocytes harbour extensive peroxidase activity, which results in relatively high background when using HRP as an enzyme for immunohistochemical studies. A considerable contribution to this is haemoglobin which is able to oxidize several molecules, including enzymatic substrates used in quantification of protein [12]. This oxidation potential has previously been used to quantify haemoproteins, including haemoglobin, since it was shown to be more sensitive than previous methods [13]. Thus, the method itself does not discriminate between infected and uninfected erythrocytes as the source of the chromogenic signal. When comparing manual and automatic quantification (Figure 2) it was noticed that: a) the assays were very comparable and, b) the automatic method did not deviate from the manual method in a systematic manner. Previous attempts to automate detection had suffered from bias at high parasite densities [11]. This method also resulted in deviations at very high erythrocyte densities (10^8^ erythrocytes/ml) (Figure 2C). Using the plate-based automatic assay, as few as approximately 2,500-3,000 erythrocytes/well (~80-100/sq mm) can be detected. However, when the lower detection limit was set it should be taken into consideration that not every single infected erythrocyte in the wells of the chamber-slides was counted manually. Since only a fraction of the area was assessed, it is more likely to miss the bound erythrocytes using microscopy at low densities compared with higher densities. In other words, precision is reduced when density is reduced, a characteristic not shared with the automatic method where the entire content of the wells is lysed.

Since the parasite digests haemoglobin and converts it to haemozoin crystals [23], it was expected that the drop in haemoglobin would result in lower chromogenic signal per erythrocyte. Although there was a trend towards a lower signal in infected erythrocytes, particularly for late-stage parasites, this turned out to be a non-significant reduction in OD. However, the signal from uninfected erythrocytes was invariably larger than that of infected erythrocytes and using the same parasite-infected erythrocytes for the standard curve as the ones used in the binding experiment is advisable. Indeed, haematin (the main component of haemozoin) has been shown to have peroxidase activity [24] and considering that most intra-erythrocytic haemoglobin has been digested in late-stage *P. falciparum*-infected cells [25], this explains the modest reduction in OD (Figure 1).

The haem-driven process can be used to fine-tune the detection of bound erythrocytes. If only a low number of malaria-infected erythrocytes bind to the adherent cells, the haem-driven chromogenic reaction can run for several minutes, improving the detection of a low number of adherent erythrocytes. The oxidation could be read continuously at 650 nm (Figure 1), but the sensitivity was increased upon acidification. As noted (Figure 1), the haem oxidation of TMB is not irreversible and as there are several types of commercially available TMB [13] it is possible to use individual modifications of the method in order to optimize it for particular purposes.

In this assay, the gravitational force was used to dissociate unbound erythrocytes as previously described [11]. However, the described assay will work with other types of washing procedures as well (e.g., using robots [8]) as long as the essence of lysing adherent erythrocytes in the wells and using the lysate to quantify the number of erythrocytes bound is maintained. Although not tested, the assay may also be applicable in assays aiming to quantify binding to recombinant proteins [3,7] instead of adherent cells. The assay requires only an ELISA reader, which most laboratories have access to.

The quantification of field isolates’ cytoadhesive properties to endothelial cells is clinically relevant. Field isolates bind to multiple endothelial receptors [3]. Addressing the binding phenotype of field isolates to both proteins [8] and glycocalyx components (e.g. HS and CS) [26,27] on endothelial cells can thus be performed without the acquisition of highly specialized equipment.

## Conclusions

This is the first automated assay to reliably quantify malaria parasites bound to adherent cells [11] without radioactively labelling parasites prior to performing the binding assay [8]. The method can be used to test binding of field isolates [3] in laboratories without specialized equipment. It has the same precision and lower limit of detection as microscopical examination and is applicable with high throughput screening of cytoadhesion.

## Abbreviations

CHO: Chinese hamster ovary
CS: Chondroitin sulfate
FBS: Foetal bovine serum
FOV: Field of view
HRP: Horseradish peroxidase
OD: Optical density
PBS: Phosphate-buffered saline
SURS: Systematic uniform random sampling
TMB: Tetramethylbenzidine
